# Randomized controlled trial protocol of health coaching for veterans with complex chronic pain

**DOI:** 10.1186/s13063-023-07113-6

**Published:** 2023-03-30

**Authors:** Sarah Crosky, Mikhaela McFarlin, Nicole Sullivan, Darren Winograd, David Litke, Robin M. Masheb, Shou-En Lu, Michelle Costanzo, Nicole Anastasides, Christina Gonzalez, Jaineel Doshi, Fiona Graff, Linda Khatib, Scott Thien, Lisa M. McAndrew

**Affiliations:** 1 Department of Educational and Counseling Psychology, University at Albany—State University of New York, Albany, USA; 2 War Related Illness and Injury Study Center, Veterans Affairs New Jersey Healthcare System, 385 Tremont Ave., East Orange, NJ 07018 USA; 3 Department of Rehabilitation Medicine, NYU Grossman School of Medicine, New York, NY 10016 USA; 4 VA Connecticut Healthcare System, 950 Campbell Ave., West Haven, CT 06516 USA; 5 Yale University School of Medicine, 333 Cedar St., New Haven, CT 06610 USA; 6 Epidemiology and Statistics, School of Public Health, Rutgers University, Piscataway, NJ 08854 USA; 7 War Related Illness and Injury Study Center, Washington DC VA Medical Center, 50 Irving St. NW, Washington, DC 20422 USA; 8 Department of Medicine, Uniformed Services University of the Health Sciences, Bethesda, MD USA

**Keywords:** Chronic multisymptom illness, Chronic pain, Health coaching, Veterans, Disability

## Abstract

**Background:**

Pain predominant multisymptom illness (pain-CMI) refers to symptom-based conditions where pain is a primary symptom. There is initial evidence that health coaching may be efficacious in treating pain-CMI because it can be tailored to the veteran’s goals and emphasizes long-term behavior change, which may indirectly impact the maintaining factors of pain-CMI (e.g., catastrophizing, poor pain control, and limited activity). This paper describes the study protocol and rationale of a randomized controlled trial that will compare the efficacy of remote-delivered health coaching in reducing disability and pain impairment for veterans with pain-CMI to remote-delivered supportive psychotherapy.

**Methods:**

This randomized controlled trial will consist of two treatment arms: remote-delivered health coaching and remote-delivered supportive psychotherapy, the active control. Each treatment condition will consist of twelve, weekly one-on-one meetings with a study provider. In addition to the baseline assessment, participants will also complete 6-week (mid-treatment), 12-week (post-treatment), and 24-week (follow-up) assessments that consist of questionnaires that can be completed remotely. The primary aims for this study are to determine whether health coaching reduces disability and pain impairment as compared to supportive psychotherapy. We will also examine whether health coaching reduces physical symptoms, catastrophizing, limiting activity, and increasing pain control as compared to supportive psychotherapy.

**Discussion:**

This study will contribute to the existing literature on pain-CMI and report the effectiveness of a novel, remote-delivered behavioral intervention.

**Supplementary Information:**

The online version contains supplementary material available at 10.1186/s13063-023-07113-6.

Chronic multisymptom illness (CMI) is an umbrella term used to describe symptom-based conditions, also called functional somatic disorders and medically unexplained symptoms. Pain-CMI refers to symptom-based conditions where pain is a primary symptom and can include fibromyalgia, chronic widespread pain, and Gulf War Illness [1, 2]. Pain-CMI is prevalent among combat veterans, especially those who deployed to the Gulf region from 1990 to 2021 [3, 4]. Approximately 30% of military personnel who deployed to the Gulf during this period have CMI, and more than 80% of veterans with CMI have chronic pain as a primary symptom[4]. Patients with pain-CMI have a significant disability [2], defined as limits to their social activities and meaningful activities (e.g., work) [5].

There is growing acceptance that symptom-based conditions, such as pain-CMI, are caused by a complex interaction between predisposing factors that place an individual at risk for the condition (e.g., genetic vulnerability), precipitating factors that trigger the onset of the condition (e.g., environmental toxic exposure, psychological trauma) and perpetuating factors that maintain the condition (e.g., inflammation, catastrophizing, activity avoidance and poor pain control) [6, 7]. Despite the medical community’s increasing understanding of pain-CMI, patients and providers report medical encounters for pain-CMI are characterized by discord. Patients and providers report disagreement about the nature of CMI [8], particularly if the precipitating and perpetuating factors are primarily psychological or physiological, and disagreement over the best treatments to address pain-CMI [9]. This disagreement causes dissatisfaction with patients reporting frustrating encounters with providers [10]. Providers, similarly, report frustration related to treating pain-CMI [11, 12].

Current evidence-based behavioral treatments are not focused on addressing the discord between patients and providers. Behavioral treatments are recommended as the first line approach for CMI management [2, 13–15], with the strongest evidence for cognitive behavioral therapy [13]. Typically, these treatments address the factors that maintain CMI. Cognitive behavioral therapy, for example, teaches skills that patients can use to directly target the cognitive and behavioral factors that maintain CMI including catastrophizing, activity avoidance, and poor perceived pain efficacy. While accumulating evidence finds behavioral treatments can work, there is also evidence that no single treatment is acceptable or efficacious for all patients with pain conditions [16]. Further, offering multiple behavioral treatments increases treatment uptake among patients with pain conditions [17]. An important opportunity to improve care for pain-CMI is to develop efficacious treatments that address the discord between patients and providers.

One promising behavioral approach that has the potential to address the discord between patients and providers is health coaching. Health coaching is a collaborative partnership between a client and a trained health coach to facilitate and encourage healthy behavior change. Health coaching is promising because it can be tailored to the patient’s beliefs about pain-CMI and their goals for treatment, thus addressing the disagreement that is common between patients with pain-CMI and their providers when using other behavioral methods.

In health coaching, the health coach and patient work together to develop a treatment plan based on the patient’s own goals for treatment. To develop this plan, the health coach works with the patient to identify discrepancies between where the patient is in various health and lifestyle areas, and where they would like to be. For example, the patient may want to improve their relationship with their daughter, improve their sleep, or eat healthier. The health coach uses motivational interviewing, goal setting, and problem-solving to help the patient achieve their goals. Health coaching can also be tailored to the patient’s beliefs about their health condition. To do this, the health coach acknowledges the patient’s beliefs about the cause of their pain-CMI and focuses on areas of agreement (e.g., the seriousness of pain-CMI and significant consequences of pain-CMI).

Health coaching largely emphasizes achievable behavior change and the establishment of long-term healthy habits, which may also indirectly impact perpetuating factors that maintain pain-CMI (e.g., catastrophizing, poor pain control, and limited activity). New research suggests that treatment may not need to explicitly target the factors underlying pain-CMI to improve these factors [18]. That is, focusing on the patient’s goals may indirectly improve factors that maintain pain-CMI. For example, reaching a goal like spending more time with one’s children may also increase activity levels. This can help the patient feel more in control of their pain [19], reduce catastrophic thinking [20], and stop the negative feedback loop that will maintain or even worsen disability.

There is initial evidence that health coaching may be efficacious and acceptable for pain-CMI. In a small pilot study where health coaches provided approximately 24 sessions to 9 patients with fibromyalgia, patients showed clinically significant improvements in health-related quality of life, pain interference, and pain severity [21]. In a larger trial, health coaching was shown to lead to reduced pain intensity and pain-related interference among patients with chronic pain [22]. However, there are still relatively few studies examining the impact of health coaching on pain-CMI, and these early studies have not used an active control condition to control for the non-specific factors, such as attention from a caring provider. Furthermore, no existing study has examined mechanisms of health coaching or has examined health coaching for veterans with pain-CMI.

The preliminary empirical and theoretical support suggests the value in determining the efficacy of health coaching for pain-CMI [18, 20–22]. The goal of the current manuscript is to present our protocol of a randomized controlled clinical trial for veterans with pain-CMI comparing remote-delivered health coaching to a remote-delivered active control, supportive psychotherapy, to reduce disability and determine mediators of treatment change.

## Methods

### Study design

The SPIRIT reporting guidelines were used to report standard protocol items for clinical trials [23]. This study is a randomized controlled trial comparing the efficacy of a remote-delivered health coaching approach in reducing disability and pain impairment among veterans with pain-CMI as compared to remote-delivered supportive psychotherapy. The study procedures were reviewed and approved by the Veterans Affairs New Jersey Health Care System (VANJHCS) Institutional Review Board on March 3^rd^, 2020 (see Additional file 1) and the full trial was registered through ClinicalTrials.gov with identifier: NCT04157101.

Participants will be enrolled in one of the two arms after completing an eligibility screening, collection of informed consent, and completion of the baseline assessment. Participants will be verbally consented into the study. Each arm consists of twelve weekly one-on-one meetings with a study provider. Participants will complete 6-week (mid-treatment), 12-week (post-treatment), and 24-week (follow-up) assessments that consist of questionnaires that can be completed online, through mail, or over the telephone with a member of the study team who is blinded to the treatment condition. There are no special criteria for discontinuing participation in the study.

### Study aims

Primary Aim 1: Determine if remote-delivered health coaching reduces (H1a) disability and (H2a) pain impairment as compared to remote-delivered supportive psychotherapy for veterans with pain-CMI. We will also assess the improvement in (H1b) disability and (H2b) pain impairment at the 24-month follow-up.

Primary Aim 2: Determine if health coaching results in more frequent clinically significant improvement in (H3) disability (20% improvement) and (H4) pain impairment (1 point improvement) as compared to supportive psychotherapy.

Secondary Aim 3: Determine if health coaching reduces (H5) physical symptoms, (H6) catastrophizing, (H7) limiting activity, and increases (H8) pain control as compared to supportive psychotherapy.

Exploratory Aim 4: Determine if the reduction in (H9) catastrophizing, (H10) limiting activity, and increases in (H11) pain control mediate the relationship between health coaching and reductions in disability and pain impairment.

### Participants

Participants will consist of 250 veterans with pain-CMI. Inclusion criteria include being a veteran who meets the Kansas City [24] definition of CMI described below in the methods section, wide-spread pain rated at least 4 on a 0 to 10-point pain bothersome scale, and activity limitations rated as at least 3 on a 0 to 10-point scale [25].

Exclusion criteria include self-report of a life-threatening condition, severe cognitive impairment, psychotic disorder, pregnancy or plans to become pregnant in the next year, or suicidal intent. Pregnancy is an exclusion as health coaching recommendations are not tailored for pregnancy (i.e., recommended changes to diet, physical activity, sleep, etc.).

### Screening and recruitment

Veterans will be recruited from anywhere in the USA. Potential participants’ contact information will be identified through the VA and DoD. We will also use referral and advertisements in both print and electronic media as potential recruitment methods. Once potential participants are identified, we will mail a recruitment letter and follow up with a phone call inviting them to participate. Once a veteran is reached on the telephone, study personnel will provide information about the study. If the veteran is interested in participating, they will be asked their personal contact information (phone numbers, address, etc.) and current location. If the veteran is in a safe place to talk (e.g., not driving), the study personnel will continue through the screening. The goal of this screen is to identify exclusion and inclusion criteria.

### Randomization and blinding

We will use an urn randomization procedure to ensure equivalent levels of our primary independent variable. Veterans will be enrolled in matched pairs based on screening pain activity limitation ratings. We will use the computer program developed for Project MATCH to generate the randomization schedule.

The study coordinator will conduct the randomization and the study providers will provide the treatment. Research staff and investigators will be blind to study assignment. To keep the staff blind, the randomization information is kept in a separate and password-protected folder on the server that is well marked. As study providers are not blind, unblinding will not occur.

### Interventions

#### Health coaching

During the 12-session remote health coaching approach, veterans will learn to develop and maintain health behaviors that coincide with their life goals. To begin, veterans will discuss their symptoms, the impact of their symptoms, and their beliefs about pain-CMI with their provider (i.e., health coach). The provider will validate the veteran’s experience and accept the veteran’s explanation for pain-CMI, which may include concerns about environmental toxins. In this example, the provider will then explain how certain military, environmental, and psychological exposures can provoke inflammation, which maintains pain-CMI.

Next, the veteran will identify discrepancies between where they are and where they want to be within 5 lifestyle areas (diet, physical activity, mind–body, sleep, and social relationships). The first half of the treatment will focus on providing education about these 5 lifestyle areas and using motivational interviewing principles to enhance the veteran’s motivation and confidence to achieve their goals. Veterans will be introduced to behavior change/health coaching principles such as SMART goal setting, habit formation techniques, behavior monitoring (e.g., food and activity logs), and problem-solving principles. Veterans will practice these behavior change skills by making small changes in each lifestyle area.

Throughout, veterans will identify goals which they may maintain from week to week or revise based on new information learned during the treatment. The major focus will be on behavior change and the development of long-term healthy habits. Therefore, behavioral consistency will be emphasized, and veterans will learn how to identify and overcome obstacles to behavior change including internal obstacles (e.g., negative thoughts and emotions) and external obstacles (e.g., busy schedules). As veterans make progress on their goals, the relationship between the veteran’s behaviors and beliefs about their pain-CMI will be continually reviewed to facilitate motivation. During the last session, veterans will develop a long-term plan to maintain behavioral changes after completion of the 12-week program; they will also identify the skills that they can utilize moving forward. Health coaching will be delivered via telephone or video teleconference. Treatment sessions will be monitored for fidelity as described below.

#### Supportive psychotherapy

Our control arm will be based on a supportive psychotherapy approach [26], which will focus on discussing weekly stressors in a supportive, non-directive way. Session content is veteran-driven, and sessions will focus on the veteran’s strengths, following the veteran’s emotional affect, and building a therapeutic alliance. Participants will be asked to generate the topic they would like to discuss for the session. For those veterans who may need additional support in identifying experiences to discuss with their study provider, they will be offered a worksheet for noting emotional events throughout their week that can be filled out between sessions (e.g., “A time when I felt stressed was …”). Veterans will be informed that the control condition is supportive and non-directive, and that providers will not engage in problem-solving. Providers will be taught to use reflective listening, clarification, empathy, and validation. The control condition consists of 12 weekly sessions delivered via telephone or video. Supportive psychotherapy is an ideal control condition in that it is thought to encompass common therapeutic factors (e.g., therapeutic alliance, emotional support), while excluding the factors specific to the health coaching (e.g., addressing discordant beliefs about pain-CMI, behavior change techniques and outside practice). Sessions will be monitored for fidelity as described below.

Throughout the course of their participation, all veterans will be allowed to begin a new treatment, continue with their existing medical regimen, or make changes as necessary. Adherence to medical protocols and participation in alternate treatments will be evaluated by a questionnaire at the assessments.

### Plan for supervision and monitoring

Providers will be licensed and credentialed mental health providers (e.g., psychologist) or mental health trainees (e.g., pre-doctoral or post-doctoral fellows) supervised by a licensed and credentialed mental health provider. Before seeing a veteran, all study providers will receive both didactic and hands-on training on health coaching, based on the VA Office of Patient-Centered Care & Cultural Transformation materials. They will also receive training with role-play in supportive counseling consistent with our supportive psychotherapy condition. Only after completing training, and with the clinical supervisor’s determination that they are competent to provide the treatment, will providers communicate with their first participant. Study providers attend individual and group supervision with a licensed and credentialed psychologist with training in health coaching, supportive psychotherapy, and differentiation between health coaching and the control condition. Trainees will also attend once-weekly individual supervision. During these sessions, the supervisor will review recordings, and discuss problems and successes, emphasizing treatment differentiation between the arms. If a study provider has poor adherence to treatment or if the licensed supervising psychologist has any concerns about competency, the provider will receive additional supervision on a one-to-one basis. Study providers will deliver both treatments.

### Treatment fidelity monitoring

We have planned a multi-step approach to ensuring fidelity, competence, and treatment differentiation for both treatments. At the core are fidelity instruments we have adapted from those used in our previous studies; we audio record sessions and code ~ 20% for fidelity. The health coaching fidelity instrument measures adherence to the active ingredients of health coaching to identify that behavioral change techniques are being used and that the provider is encouraging the veteran to choose their own behavioral goals. The supportive psychotherapy fidelity instrument measures adherence to the use of the active ingredients of this treatment (e.g., empathy) as well as identifying if there is any inappropriate drift into health coaching through scoring of a set of differentiation variables. This will enable us to ensure that active ingredients of the two approaches remain separate. We will intervene if there is poor adherence to treatment or low competency. NIH’s Behavioral Change Consortium's treatment fidelity framework and Perpletchikova recommendations [27, 28] on treatment integrity guide our fidelity monitoring.

### Minimizing attrition

We will schedule appointments at a time most convenient for the Veteran (including evenings). We will likely make reminder phone calls approximately 24 h prior to their session. It is our experience that missing sessions, no matter what the reason, often leads to attrition. Therefore, the study staff will try to call a Veteran within 24 h if they miss a session.

### Adverse event reporting

Veterans will be asked about adverse events at all sessions and assessments. Adverse events will be captured and reviewed by a study clinician. Adverse events will be reported to their IRB annually and serious adverse events are reported sooner. There are no planned formal stopping rules for the trial nor interim analyses as there are no anticipated problems that are detrimental to the participants. Due to the low risk of the intervention, there is not a Data Monitoring Committee.

### Measures

#### Primary outcomes

Consistent with current recommendations, our primary outcomes will include a general measure of disability and a condition-specific measure of pain impairment [29]. The World Health Organization Disability Assessment Schedule (WHO-DAS 2.0) [5, 30] is a 40-item general measure of disability and assesses two underlying constructs: activity limitations and deficits in social integration. The items of the WHO-DAS have a factor loading on a composite score of 0.82 to 0.98. The WHO-DAS has been found to have high reliability and validity. A 20% improvement will be considered clinically significant [13].

Pain interference will be assessed using the Brief Pain Inventory (BPI). The BPI is an 11-item measure of pain severity and interference. The BPI has been recommended as a core measure of clinical trials due to its reliability, validity, and responsiveness to clinical intervention [29, 31]. A change of 1 point for the interference subscale will be considered clinically significant.

#### Secondary outcomes

The Patient Health Questionnaire (PHQ-15) [32] is a reliable and valid measure of physical symptoms and is responsive to change. Perceived control beliefs will be measured by the Illness Perception Questionnaire-Revised (IPQ-R) [33]. The IPQ-R measures a broad range of illness beliefs, including the severity of illness consequences, chronicity, and controllability of illness. The IPQ has been found to be reliable and valid among patients with CMI, predictive of outcomes, and responsive to clinical intervention [34–36]. Catastrophizing will be measured by the Pain Catastrophizing Scale (PCS). The PCS is a 13-item measure of the degree of pain catastrophizing. The PCS [7, 37–39] is reliable, valid, predictive of outcomes, and responsive to clinical intervention. Limiting behaviors will be assessed with the Behavioral Response to Illness Questionnaire (BRIQ) which measures behavioral responses to CMI. The BRIQ has four subscales: limiting behaviors, all or nothing behaviors, practical support, and emotional support. The BRIQ is reliable, valid, and predicts the onset of CMI [40].

### Participant characterization and screening instruments

The National Academy of Medicine recommends the Kansas Case Definition [24] to identify CMI. To meet the criteria, veterans endorse moderately severe and/or multiple symptoms in at least 3 of 6 domains. Self-reported diagnosis of chronic conditions (e.g., cancer) that can produce diverse symptoms (e.g., pain) or might interfere with the respondents’ ability to accurately report their symptoms is excluded. We use a modified list that excludes individuals with conditions that may account for CMI, but which includes individuals with conditions that are common in an aging population (e.g., diabetes) or less likely to account for CMI to improve generalizability.

Pain-predominant CMI is CMI where pain is a primary symptom. Fibromyalgia is the best-known example of this, but veterans with CMI and pain who do not meet the criteria for fibromyalgia may also have pain-CMI. To ensure pain is a primary symptom, we will assess that the pain is bothersome (at least a 4 on a 0–10 point scale) and impairing (at least a 3 on a 0–10 point scale) [25].

The Columbia-Suicide Severity Rating Scale (C-SSRS) is the gold standard for assessment of intensity of suicidal ideation, plans and preparation for suicidal behavior [41]. The C-SSRS will be used at screening and the end of treatment. 

We will also ask about basic demographic information including medical conditions, treatment, sex, age, marital status, etc. We will confirm this information through a medical chart review.

The twenty-item National Center for Post-Traumatic Stress Disorder (PTSD) Checklist (PCL-5) [42], will be used to assess PTSD. The PCL-5 asks participants the degree by which they have been bothered by a symptom over the last month. The PCL-5 is a valid and reliable measure of PTSD symptoms.

The Patient Health Questionnaire-9 (PHQ-9) [43] is a 9-item self-report questionnaire that assesses the frequency of depressive symptoms over the past 2 weeks. The total score of the PHQ-9 has also been used as indicative of depression severity and is sensitive to clinical change.

### Measures of the quality of treatment delivery

#### Treatment fidelity: veteran’s beliefs and goals

To ensure our treatment addresses the veteran’s beliefs and goals, we will include a measure of concordance of illness beliefs (if the veteran perceives they agree with the study provider about Pain-CMI) [44], and a measure of meeting personal goals for treatment. We will also observe and code the treatment sessions for fidelity.

#### Treatment fidelity: health behavior change

To ensure health coaching directly emphasizes behavior change and that the control condition does not, we will include a measure of diet [45], sedentary behavior [46], sleep [47], perceived stress [48], and social support [49].

#### Patients’ experience with treatment

We will ask veterans about their experiences with treatment using a short, validated satisfaction measure [50], the 2-item Patient Global Impression of Change (PCIG) [51] which asks patients about their perceived improvement, and a measure of participants’ perception of their relationship with their study provider [52].

### Statistical analysis plan

All statistical analyses will be performed on an intention-to-treat basis. For each test, the statistical significance will be defined by *p* < 0.05. Bonferroni correction will be applied for multiple testing, where appropriate.

#### Analysis plan for Aim 1

Our first aim is to determine if remote-delivered health coaching reduces (H1) disability and (H2) chronic pain impairment as compared to supportive psychotherapy for veterans with pain-CMI. To address Aim 1, we will use mixed model analysis. Specifically, the statistical model will include each of the outcome variables, disability (measured by WHO-DAS 2.0) and chronic pain impairment (measured by BPI), as the dependent variable, and treatment condition (remote-delivered health coaching vs. active control), time (pre, mid, post-treatment, and follow-up), and treatment by time interactions as the independent variables. Random intercept will be included in the model to account for the intra-subject correlation between repeatedly measured outcomes. Covariates such as age, gender, baseline level of disability severity, and baseline variables that showed a significant difference (*p* < 0.1) will be controlled in the analysis. Improvements from pre- to post-treatment (H1a, H2a) and pre-treatment to follow-up (H1b, H2b) will be compared between treatment groups using linear contrasts.

#### Analysis plan for Aim 2

Our second aim is to determine the difference in response rate. We hypothesize that more veterans with pain-CMI randomized to remote-delivered health coaching will have a clinically significant improvement in (H3) disability and (H4) chronic pain impairment as compared to supportive psychotherapy after treatment. To address Aim 2, we will use logistic regression analysis. The dependent variable will be binary (yes/no to [H3]) if the improvement in disability, measured by WHO-DAS 2.0, exceeds 20% from the baseline, and yes/no to (H4) if the improvement in chronic pain impairment, measured by BPI, meets or exceeds 1 point improvement from the baseline). The study arm will be an independent variable in the model. The difference in the treatment effect between remote-delivered health coaching vs. supportive psychotherapy will be assessed by estimating and testing the regression coefficient of the treatment condition variable in the model. Covariates including but not limited to age, gender, and baseline level of disability severity will be controlled in the analysis.

#### Analysis plan for Aims 3 and 4

Our third aim is to determine if health coaching reduces (H5) physical symptoms [32], (H6) catastrophizing [38], (H7) limiting activity [40], and increases (H8) pain control [33] as compared to supportive psychotherapy. For Aim 3, each variable in H5-H8 will be compared using the mixed model analysis described in Aim 1. Our exploratory Aim 4 is to determine if the reduction in (H9) catastrophizing, (H10) limiting activity, and increases in (H11) pain control mediate the relationship between health coaching and reductions in disability and pain impairment. To address H9–H11, we will perform mediation analysis following the recommendations of Baron and Kenny [53].

#### Statistical treatment for missing data

Missing data are common in longitudinal studies. To address missing data, when the data can be assumed to be "missing at random" (MAR), we will use the likelihood-based statistical methods (i.e., mixed model and logistic regression analyses) [54]. To address missing data when data can not be assumed to be MAR, we will perform a series of sensitivity analyses assuming not-missing-at-random (nMAR) and/or a mix of nMAR and MAR, using multiple imputation procedures [55, 56]. We will also explore methods to model the missingness mechanism and apply the methods of selection models [57] or use the pattern-mixture models [58] such as the tipping-point approach or control‐based pattern imputation approach [59].

#### Treatment fidelity check

Our aims are predicated on the assumption that health coaching addresses veterans’ beliefs and goals and changes health behaviors. To check that health coaching accomplished this we will conduct exploratory analyses to determine if health coaching has a greater concordance of illness beliefs, reached veteran’s goals for treatment, improves diet, physical activity, sleep, and social support, and reduces stress. Each variable will be compared using the mixed model analysis described in Aim 1.

### Power and sample size considerations

We will enroll 250 veterans with CMI (*n* = 125/group) and randomize them to either remote-delivered health coaching or supportive psychotherapy control at a 1:1 ratio. To account for 15% attrition, we based our power/sample size analysis using *n* = 106/group. This sample size will allow us to test a minimal effect size, in terms of Cohen’s *d* = 0.39 (a moderate effect size per Cohen) when we test the primary outcomes (H1-H2) in Aim 1 and secondary outcomes in Aim 2 with 80% power and alpha = 5% (two-sided).

## Discussion

The proposed protocol will examine the efficacy of remote-delivered health coaching in reducing disability and pain impairment for veterans with pain-CMI as compared to remote-delivered supportive psychotherapy. Health coaching offers a patient-centered approach that focuses on veteran goals while potentially addressing the maintaining factors of pain-CMI. Thus, health coaching may not only improve the disability associated with pain-CMI, but also address the disagreement between patients and providers on the nature and treatment of pain-CMI. Health coaching can also be delivered remotely and by peer-health coaches and bachelor and master level health coaches, thus increasing accessibility to care among this population.

In developing this study, we considered and decided against a comparative effectiveness study comparing health coaching to CBT. There is preliminary data [21] that health coaching may be efficacious for pain-CMI. However, there has never been a clinical trial comparing health coaching to an active control such as supportive psychotherapy for pain-CMI. Considering that CMI is reactive to placebo interventions [60], it will be difficult to interpret the results of a comparative effectiveness trial without knowing if health coaching is more effective than a placebo.

### Limitations

This study will have several limitations. First, the study relies on self-report data for measuring disability and pain impairment. While self-report outcomes are important, collateral information regarding functioning, symptomatology, and pain impairment would enrich the data. However, due to the remote delivery of the intervention, the present study design does not allow for measuring additional information such as biomarkers of inflammation, or other assessments of disability. Additionally, we cannot know if the results of a remote-delivered intervention will carry over to in-person health coaching delivery. Similarly, the health coaches administering the treatments in our study will be psychologists or psychology trainees, and thus it is unclear how this will translate to bachelor or peer health coaches for downstream implementation. Lastly, the current study is specific to veterans with pain-CMI who receive care within the VA system, which limits the generalizability of the results and knowledge of the feasibility of such an intervention for the wider civilian population or non-VA settings.

### Possible implications

We hypothesize health coaching will be an efficacious option for treating pain-CMI within the veteran population. If this is shown to be the case, health coaching would provide an additional evidence-based behavioral approach to effectively manage complex pain and other chronic physical symptoms. Health coaching may be particularly appealing for patients who are dissatisfied with current approaches as it may mediate patient-provider disagreement by personalizing the intervention to the patient’s beliefs and goals. This customized behavioral focus may help patients feel more motivated to adhere to treatment, gain control over their lives, and subsequent control over their pain.

This study also has theoretical implications. Current behavioral treatments directly target mechanisms thought to perpetuate chronic pain, irrespective of the patient’s goals. For example, teaching patients to challenge catastrophizing beliefs or gradually increase activity to reduce limiting activity. While efficacious for many, not all patients are willing to engage with treatments that do not address their goals. If we find that by helping patients meet their personalized goals, health coaching can indirectly address the mechanisms thought to perpetuate pain, this will improve our theoretical understanding of how behavioral treatments work.

Finally, this study also has implications for treatment accessibility. Pain-CMI can make it difficult to attend treatment sessions given limited mobility and increased disability. Adapting health coaching as a remote-delivered modality may enhance accessibility to veterans who might otherwise have trouble accessing in-person treatment. If found to be effective, we hope that our tailored intervention might serve as a model for delivering health coaching to patients with pain-CMI to help reduce disability and pain impairment.

## Supplementary Information

**Additional file 1.**

## Data Availability

Requests for access to the final dataset can be made to Lisa M. McAndrew and requests must be approved by the VA.
