# Supplementary material for: Randomized controlled trial protocol of health coaching for veterans with complex chronic pain
Source: Trials. 2023 Mar 30;24:239. doi: 10.1186/s13063-023-07113-6 (PMC10061706; doi:10.1186/s13063-023-07113-6)
Supplement: Supplementary file 1 — Additional file 1. [file 13063_2023_7113_MOESM1_ESM.pdf]

VANJHCS IRB OFFICE USE ONLY
VANJHCS IRB
MARCH 02 2020
APPROVED

Version #: 1 ***Version Date: 02/11/2020***

Title of Study: ***Health Coaching for Chronic Multi-symptom Illness (CMI)***

Principal Investigator: Lisa McAndrew, PhD IRB#: 01505

Subject Name: ________________________________ **Date:**_______________

CONCISE SUMMARY

This is a research study to find out if remote-delivered health coaching is beneficial in reducing the disability and impairment in Veteran's caused by chronic pain. To enroll in this study, you must be a Veteran that meets the Kansas City (Steele) definition of Chronic Multi-symptom Illness (CMI); have widespread pain and report activity limitations. You will be screened for the above prior to enrollment. You will be randomized to one of two arms of the study: **Health Coaching** or **Attentional Control**. Neither group is a replacement for clinical care. Neither group is a treatment for suicidal thoughts.

All the sessions, no matter which group you are assigned to, will be delivered via phone or VA Video Connect (VVC). Each session will take approximately 1 hour and there are 12 sessions. Questionnaires will be filled out during the screening session; at the baseline session; 6 weeks after the sessions begin; 12 weeks after the sessions begin and 24 weeks after the sessions begin. The questionnaires can be completed in paper, on the computer or over the phone with a study team member. There are approximately between 17 and 20 questionnaires be completed at different timepoints during the study. If you are interested in learning more about this study, please continue reading below. *Veterans who are pregnant or plan to become pregnant are excluded from participating in this study.*

Read the information below and discuss it with family and friends if you wish. Ask one of the study staff if there is anything that is not clear or if you would like more details. Take your time to decide. If you do decide to take part in this study, your signature on this consent form will show that you received all the information below, and that you were able to discuss any questions and concerns you had with a member of the study team.

The PI of this study is Lisa McAndrew, PhD and the research is being supported by the Department of Veteran Affairs: Rehabilitation Research & Development Service (RR&D).
Up to 250 Veterans will take part in this study.

BACKGROUND & PURPOSE

Chronic pain is one of the most extensive health care issues facing our society with "severe impacts on all aspects of the lives of its sufferers". Pain-CMI (e.g., fibromyalgia) is a particularly debilitating and treatment-resistant chronic pain condition and a presumptive service-connected condition for Veterans who deployed to the Gulf region from 1990-2010.
The VA/DoD Guidelines recommend non-pharmaceutical treatments (e.g., cognitive behavioral therapy) for Pain-CMI and find evidence they improve disability (typically) by targeting the mechanisms underlying Pain-CMI (e.g., low perceived pain control, catastrophizing and limiting activity). While no

single treatment is (or is likely to be) acceptable, available and effective for all Veterans, having a wide array of treatment options facilitates Veterans' acceptance of non-pharmaceutical approaches.

Health coaching is an appealing potential approach to improve the disability of Pain-CMI because it is personalized to the Veteran's unique goals and beliefs about Pain-CMI and it will be widely available as the VA is rolling out health coaching for other conditions. The goal of this proposal is to understand if health coaching is also effective for Pain-CMI. With this research study, we hope to learn how helpful remote-delivered health coaching is in reducing the disability and impairment caused by Veterans' chronic pain. We will be comparing results to Veterans who are receiving remote-delivered attentional control. Both health coaching and the attentional control condition are not replacements for standard mental health treatment or usual care. We strongly encourage you to continue with the care you are already receiving from your providers and you can begin any new treatment you would like while participating in this study.

DURATION OF THE RESEARCH

This research study is expected to take approximately 5 years. Your individual participation in the project will take 6 months with sessions of 1 per week for 12 weeks and follow-up in 24 weeks.

PROCEDURES

You will complete the informed consent process, which includes reviewing the informed consent document and Health Insurance Portability and Accountability Act (HIPAA) form with a study team member. This process may take place over the phone with a study team member.
We will collect the contact information (name, phone number, and mailing address) of your primary care and mental health providers. This information may be obtained and confirmed either from your medical record or through a form you will complete. We are getting this information so we may let your primary care and mental health providers know about your participation in this study. This will enable your providers to let us know of any health concerns s/he may have about your participation. We highly encourage you to reach out to your providers to discuss your participation in the study. We may also contact your primary care or mental health provider if we have any medical concerns about you during the study.

- After you complete the informed consent process, you will be asked to fill out a questionnaire packet. Completing this questionnaire packet can be done either over the phone, over the internet through Qualtrics, or mailed back to us in a prepaid envelope. You are free to skip any questions that you prefer not to answer.
- You will also be asked to complete questionnaire packets at 4 different timepoints:
    - at the beginning of the study,

  - after the 6th session of either health coaching or attentional control,
  - post treatment (after the 12th session of either health coaching or attentional control),
  - and 24 weeks after the first session.
- In this study, you will be randomized (like flipping a coin) into one of two groups; one group will receive 12 sessions of remote-delivered health coaching and the other group will receive 12 session of remote-delivered attentional control. Health coaching and attentional control can be delivered through the VA's Video Connect (VVC) technology or by telephone. Sessions will last ~1 hour and may be audio recorded.

- Throughout the course of the study you can continue to receive all standard of care treatments or any new treatments recommended by your healthcare providers.

  - If you are in the **health coaching** group you will be mailed a workbook and during your time with the Study Provider, you will discuss various health topics. Health topics may include food and diet, social connection and relationships, working the body, sleep and recharge, and stress management. You will also make goals for each topic discussed and be taught tips to assist you in reaching your health goals. You will have 12 weekly health coaching sessions either through VVC or over the phone with a Study Provider. Each session will last about an hour and may be recorded. The study provider may make changes to the treatment (e.g., change the order information is presented) if it is in your best interest. We will enter notes into your medical record after these sessions.

    During the sessions, you will also be asked to follow some basic rules (i.e., not having anyone else in the room and shutting off other telephones so there will be no distractions). You will be able to receive any care you are currently receiving from any VA or non-VA providers or make any changes to your care you would like to make.

  - If you are in the **attentional control** group, you will have 12 weekly attentional control sessions either through VVC or over the phone with a Study Provider. Each session will last about an hour and may be recorded. During your time with the Study Provider, you will have the opportunity to discuss any concerns you may have, which could include stressors or successes you experience, etc. You will be provided discussion starter forms that you may use. Sessions are designed to be supportive you will not discuss problem-solving or behavioral change techniques during these sessions. Attentional control is not the same as standard mental health treatment or usual care which could include problem-solving and behavioral change. If you are interested in receiving standard mental health treatment during your participation in this study, you may do so.

- There are other treatment options available to help manage chronic pain, like cognitive-behavioral therapy. Please contact your medical care providers if you are interested in learning more about such options.

  During the sessions, you will also be asked to follow some basic rules (i.e., not having anyone else in the room and shutting off other telephones so there will be no distractions). You will be able to receive any care you are currently receiving from any VA or non-VA providers or make any changes to your care you would like to make.

Suicidal thoughts: Neither group is a treatment for suicidal thoughts. If you have thoughts of hurting yourself, we will talk with you about these thoughts and talk with your usual care provider to ensure you are safe. We encourage all Veterans with thoughts of hurting themselves to receive treatment to prevent suicide. If you experience an increase in thoughts of wanting to hurt yourself, we will consult your usual care provider and evaluate if participating in the study is helpful to you right now.

- Voice Recordings: Only with your permission, Study Providers may audio record sessions for research purposes only. You may decide to change your mind about allowing audio-recording the session at any time. The information will be accessed by the PI and her research study team. Audio recordings will be downloaded and stored on a secure shared network folder created by the Information Resource Management (IRM) in East Orange, NJ. Any audio recording device will be stored in a locked filing cabinet; the audio recordings will be stored on a secure shared network folder and then disposed of as per VA's policy.

  I have read and understand the above statement and I consent to the use of my voice as specified for the above-described purpose(s).

  __________ Initials

- Follow-up questionnaires will be completed at three additional time points:
  - after the 6th session of either health coaching or attentional control,
  - post treatment (after the 12th session of either health coaching or attentional control)
  - 24 weeks after the first session

- You can refuse to participate in any portion of the study and still take part in the rest of the study.

- You will interact with Study Coordinators and Study Providers.

- Each session will take place weekly at a time of your choosing.

Veterans who are pregnant or plan to become pregnant are excluded from participating in this study.

PARTICIPANT RESPONSIBILITIES

During your participation in the research, we ask that you do the following:

- Keep your study appointments. If it you miss an appointment, please contact the investigator or research staff to reschedule as soon as you know you will miss the appointment.
- Tell the investigator or research staff if you believe you might be pregnant.
- Complete your questionnaires as instructed.
- Ask questions as you think of them.
- Tell the investigator or research staff if you change your mind about staying in the study.
- While participating in this research study, do not take part in any other research project without approval from the investigators. This is to protect you from possible injury. Taking part in other research studies without first discussing it with the investigators of this study may invalidate the results of this research, as well as that of the other studies.

POSSIBLE RISKS OR DISCOMFORTS

Any procedure has possible risks and discomforts. The procedures in this study may cause all, some, or none of the risks or side effects listed. Rare, unknown, or unexpected risks also may occur.

- Emotional Stress: It is possible that some participants could find some of the questions in the questionnaire packets upsetting. It is also a possibility that some participants may find the health coaching or attentional control sessions upsetting.

    - If you are experiencing emotional distress you may contact the National VA CRISIS LINE at**1- 800-273-8255**. Someone will be available to talk to you 24 hours a day, every day of the year.

    - You may also call the NJ War Related Illness and Injury Study Center (WRIISC) and ask to speak with a mental health provider who is part of our study team at **1-800-248-8005**. Mental health providers are typically (but not always) available Monday – Friday 8am to 4 pm eastern standard time.

- Loss of Confidentiality: There is a risk that study information (data) could be connected to your name. This is called “loss of confidentiality.” Data collected from the study, including audio recordings of sessions, will be maintained on the VA server with access limited to the study team.

The methods and procedures used for data storage and security are consistent with those adopted by VA NJHCS.

If you complete your questionnaires online, there are additional risks of loss of information. This is minimized by using a VA approved company that follows all the VA rules to keep your data secure. You may choose to complete your questionnaires on paper to reduce this risk.

- As part of health coaching, you may decide to make changes to your daily activities (e.g., starting a new diet, doing physical activity, increase social activities). If and how you make these changes is your choice. There could be risks associated with any changes you make to your daily life. You should consult with your primary care provider or other medical provider to minimize any risks and address any concerns you may have about changes you may wish to make. We may also contact your health care provider.

    - You will be encouraged to eat a diet consistent with the Mediterranean diet. This is a high fiber diet and could cause bloating, gas, or indigestion.

    - You will be encouraged to increase social activities of your choosing; there could be risks with any change that you make.

    - You will be encouraged to slowly increase physical activity. Physical activity may cause an increase in heart rate, an increase in blood pressure, shortness of breath, general fatigue, and in some cases muscle soreness or injury to the muscle, bone, or joint. Exercise may cause you to experience a serious cardiac event, an arrhythmia, or chest pain. The possibility of experiencing a serious cardiac event has been estimated to be about 6 per 10,000 in exercising adults.

There is always a chance that any procedure can harm you. The procedures in this study are no different. In addition to the risks described above, you may experience a previously unknown risk or side effect.

Risks of the usual care you receive are not risks of the research. Those risks are not included in this consent form. You should talk with your health care providers if you have any questions about the risks of usual care.

POTENTIAL BENEFITS
There are no direct benefits to you from your taking part in this research study. However, the information we get from this study might help us treat future patients.

CONFIDENTIALITY

- Taking part in this study will involve collecting information, including private information about you. Private information may include the following: age, address, health information, (e.g., diagnoses, symptoms), treatment recommendations, your voice, and last four digits of your social security number. Data collected may include the following: answers from the questionnaires you complete and recordings from sessions with your study provider.

- The methods and procedures used for data storage and security are consistent with those adopted by VA. This information will be protected in the following ways:

    - You will be assigned a unique identification number. All data is coded with this unique identification number. There is an electronic “link” that connects your name and the unique identification number. The “link” will permanently reside in a password protected electronic file on the VA secure server created by the IRM in East Orange, NJ. All data from the questionnaires and recordings will be inputted to a database with your unique subject number. No personal information, e.g. your name, will be inputted with your data. Only members of the study team will have access to this “link,” the shared network folder, and its electronic files.

    - If you decide to complete your questionnaires on the Qualtrics website, your data will be temporarily stored on the secured Qualtrics server. This data will be identified only with your unique identification number, which you will be assigned. The data will be transmitted to us through encryption methods that are VA approved. This data will then be uploaded onto our secured shared network folder within the secure VA server created by the IRM in East Orange, NJ.

    - All paper copies will be kept in a locked cabinet in a locked office. Only members of the study team will have access to these cabinets.
    - Some of the data analysis will be completed by our collaborator at Rutgers University School of Public Health.

Information about you will be combined with information from other people taking part in the study. We will write about the combined data we have gathered. Any talks or papers about this study will not identify you.

The information collected for this study will be kept confidential. We will include information about your study participation in your medical record. There are times when we might have to show your records to other people. For example, someone from the

Office of Human Research Protections or other federal, state, or international regulatory agencies
VA Office of Research Compliance
VANJHCS IRB and Research and Development Committees
Study monitors that may look at or copy portions of records that identify you.

A description of this clinical trial will be available on http://www.ClinicalTrials.gov as required by U.S. Law. This website will not include information that can identify you. At most, the website will include a summary of the results. You can search this website at any time.

COSTS TO PARTICIPANTS AND PAYMENT

Cost to Participants*:*
You will not be charged for any treatments or procedures that are part of this study. If you usually pay co-payments for VA care and medications, you will still pay these co-payments for VA care and medications that are not part of this study.

Tax law may require the reporting of the amount of payment you received for participating in research to the Internal Revenue Service (IRS) or other agencies, as applicable. Generally, this reporting would take place if you received payments that equal $600 or more in a calendar year. You would be responsible for the payment of any tax that may be due on this additional income.

Payment Offered for Participation:
You will be compensated for your participation in this study up to a total of $275 for completing and returning questionnaires as follows:

- Baseline questionnaire = $50
- 6-week mid treatment questionnaire = $50
- Post-treatment (12-week) questionnaire = $100
- 24-week follow-up questionnaire = $75

You will sign a voucher giving us permission to pay you. You may be asked for your social security number on these vouchers so you can be paid. Due to limitations in the Financial Management System, payments made to you through Austin Financial Services Center generate an Internal Revenue Service Form 1099 regardless of amount.
The voucher should be mailed with the completed questionnaires in the pre-paid envelopes provided. You should receive a check in about 6-8 weeks. If you are at the VA NJHCS in person, you may receive cash. If you have direct deposit setup, you may receive payment directly to the account you specified.

MEDICAL TREATMENT AND COMPENSATION FOR INJURY

Every reasonable safety measure will be used to protect your well-being. If you are injured as a result of taking part in this study, the VA will provide necessary medical treatment at no cost to you unless the injury was due to your not following the study procedures.

If you should have a medical concern or get hurt or sick as a result of taking part in this study, call:

DURING THE DAY: Lisa McAndrew, PhD or Ms. Nicole Anastasides
Phone: **973-676-1000 Ext. 1-1167**

AFTER HOURS:

VA NJHCS Emergency Room
Phone: **973-676-7236 or 973-676-1000 Ext. 1-1222**

Emergency and ongoing medical treatment will be provided as needed.

You do not give up any of your legal rights and you do not release the VA from any liability by signing this form.

PARTICIPATION IS VOLUNTARY

It is up to you to decide whether or not to take part in this study. If you decide to take part, you may still withdraw at any time. If you do not wish to be in this study or you decide to leave the study early, you will not lose any VA benefits to which you are entitled. If you don't take part, you can still receive all usual care that is available to you at the VA. Your decision not to take part will not affect the relationship you have with your doctor or other staff, and it will not affect the usual care that you receive as a patient.

If you decide to withdraw from the study prior to completion, data already collected prior to your withdrawal may still be reviewed and analyzed.

RIGHT OF INVESTIGATOR TO TERMINATE PARTICIPATION

The Principal Investigator may stop you from taking part in the study at any time if she believes that it is in your best interest and/or the best interest of the study.

PERSONS TO CONTACT ABOUT THIS STUDY

If you have any questions, complaints or concerns about your rights as a study participant, you should contact the **Patient Representative at 973-676-1000 extension 3399**.

If you want to make sure this is a valid VA study, you may contact the VANJHCS Institutional Review Board (IRB). This is the Board that is responsible for overseeing the safety of human participants in this study. You may call the **VANJHCS IRB at 973-676-1000 ext.1- 2778** if you have questions, complaints or concerns about the study or if you would like to obtain information.

PAYMENT TO INVESTIGATORS
Lisa McAndrew, PhD is being paid by the Department of Veteran Affairs: Rehabilitation Research & Development Service (RR&D) to conduct this study.

FUTURE USE OF DATA AND RE-CONTACT

With your permission, we would like your data or health information to be reused later for additional research purposes. If you agree, your data will be stored in a VA data repository, for future use. All the data in the repository will be coded and will reside on a secure server administered by the Information Resource Management (IRM). Future analysis of data stored within the data repository will only happen after further Institutional Review Board and/or other applicable approvals to ensure the protection of your individual privacy. Please read the statement below and check one box.

- I give my permission for Veterans Health Administration (VHA) to store my data collected from this study, including health information, in a research data repository.

  ☐ YES ☐ NO _______Initials

With your permission, we may also contact you in the future to invite you to take part in additional studies in the future. Please read the statement below and check one box.

- I give my permission for the Principal Investigator or a member of the study team to contact me to ask me to take part in additional studies in the future.

  ☐ YES ☐ NO _______Initials

AGREEMENT TO PARTICIPATE IN THE RESEARCH STUDY

Dr./Mr./Ms.____________________________ has explained the research study to you. You have been told of the:

- risks or discomforts and
- possible benefits of the study.

You have been told of:

- other choices of treatment available to you.

You have been given the chance to ask questions and obtain answers.

By signing this document below, you voluntarily consent to participate in this study. You also confirm that you have read this consent, or it has been read to you. You will receive a copy of this consent after you sign it. A copy of this signed consent will also be put in your medical record.

**I agree to participate in this research study as has been explained in this document.**		
**Participant's Name**	**Participant's Signature**	**Date**
**Witness Name***	**Witness Signature**	**Date**

***As per New Jersey State Law, the witness cannot be an authorized representative or the researcher or a representative of the researcher and can attest the requirements of informed consent have been satisfied.**
